# Trehalose and glucose levels regulate feeding behavior of the phloem-feeding insect, the pea aphid *Acyrthosiphon pisum* Harris

**DOI:** 10.1038/s41598-021-95390-z

**Published:** 2021-08-05

**Authors:** Guang Wang, Jing-Jiang Zhou, Yan Li, Yuping Gou, Peter Quandahor, Changzhong Liu

**Affiliations:** 1 College of Plant Protection, Gansu Agricultural University, Lanzhou, 730070 China; 2 Biocontrol Engineering Laboratory of Crop Diseases and Pests of Gansu Province, Lanzhou, 730070 China; 3 State Key Laboratory of Green Pesticide and Agricultural Bioengineering, Ministry of Education, Guizhou University, Huaxi District, Guiyang, 550025 China

**Keywords:** Animal physiology, Entomology

## Abstract

Trehalose serves multifarious roles in growth and development of insects. In this study, we demonstrated that the high trehalose diet increased the glucose content, and high glucose diet increased the glucose content but decreased the trehalose content of *Acyrthosiphon pisum*. RNA interference (RNAi) of trehalose-6-phosphate synthase gene (*ApTPS*) decreased while RNAi of trehalase gene (*ApTRE*) increased the trehalose and glucose contents*.* In the electrical penetration graph experiment, RNAi of *ApTPS* increased the percentage of E2 waveform and decreased the percentage of F and G waveforms. The high trehalose and glucose diets increased the percentage of E2 waveform of *A. pisum* red biotype. The correlation between feeding behavior and sugar contents indicated that the percentage of E1 and E2 waveforms were increased but np, C, F and G waveforms were decreased in low trehalose and glucose contents. The percentage of np, E1 and E2 waveforms were reduced but C, F and G waveforms were elevated in high trehalose and glucose contents. The results suggest that the *A. pisum* with high trehalose and glucose contents spent less feeding time during non-probing phase and phloem feeding phase, but had an increased feeding time during probing phase, stylet work phase and xylem feeding phase.

The understanding of insect feeding behavior is important in insect pest management. Previous studies have shown that insect feeding behavior is strongly influenced by biotic and abiotic factors^1–5^, as well as by the change in its physiology status, host plant nutrition and species^6^, and resistance to pesticide^3,7^. Aphids use their stylets to obtain nutrients from sieve tubes of plant tissue, and ingest passively on the phloem, driven by the pressure in the sieve tubes, and actively on the xylem, intercellular apoplastic and epidermal^8^. The stylet penetrates into the plant tissue and forms a stable food channel by secreting saliva to ingest plant sap^9^. The electrical penetration graph (EPG) has been used to monitor stylet activity, saliva excretion and food ingestion during aphid feeding and to record stylet tip positions and activities as different EPG waveforms^3,7,10^. The EPG waveform np, C and E1, represent non-probing, intercellular apoplastic stylet pathway and salivation into phloem sieve elements, respectively, at the beginning of the phloem phase^4,7^. While the EPG waveform E2, G and F are correlated with passive phloem sap uptake from sieve element, active intake of xylem sap and derailed stylet mechanics, respectively^4,7^. Interestingly, it was reported that aphids aposymbiotics (the disruption of the endosymbiotic bacteria *Buchnera aphidicola*), pesticides and pathogen *Pandora neoaphidis* also affected the feeding behaviors of piercing-sucking insects^3–5,7^. However, the studies on the effects of body sugar levels on aphid feeding behavior and associated EPG waveforms are very limited.

Sugar such as trehalose is widely present in bacteria, fungi, insects and plants, and is formed by two glucose molecules linked by an α–α bond^11,12^. It is mainly present as a non-reducing disaccharide in insect hemolymph, and typically occurs at a high concentration; whereas glucose may occur together with trehalose but at a significantly lower concentration^13^. Trehalose plays important roles in the growth, development^14,15^, flight^16,17^, feeding^18^, overwinter and diapause^19^ of insects. Simpson and Raubenheimer (1993)^20^ suggested that hemolymph trehalose level reflects the nutritional status of the insect and may serve a role in regulating food choice and nutrient consumption. Dietary nutrient levels on gluconeogenesis in *Manduca sexta* was positively correlated to hemolymph trehalose levels^21^, and the ratio of carbon to nitrogen from carbohydrate absorption affected the growth and development of *Acyrthosiphon pisum*^2,22^. Trehalose serves multifarious roles in regulating insect feeding behaviour and nutrient intake such as facilitating carbohydrate absorption, being a source of energy, and a component of a feedback mechanism^11,12,23^. However, the feedback mechanism of high trehalose contents on the feeding behaviours of piercing-sucking insects have not been clarified yet.

It is well known that trehalose-6-phosphate synthase (TPS) and trehalase (TRE) can directly or indirectly affect trehalose content and feeding behavior^15,18,24,25^. Knockdown of TRE genes increased the trehalose content and reduced the food intake of *Spodoptera exigua*^26^, while knockdown of *TPS* reduced the trehalose contents but did not affect the feeding behaviors of *Nilaparvata lugens*^15^, *Bactrocera minax*^25^ and *Leptinotarsa decemlineata*^18^. We have recently shown that RNAi of *ApTPS* and *ApTRE* effected the chitin metabolism of *A. pisum*^27^. A more comprehensive study of trehalose level and feeding behavior is necessary because sugars are the main components of plant sap that aphids feed on. However, the effects of *TPS* and *TRE* on the feeding behavior and the detailed relationships between the trehalose content and feeding behaviors of *A. pisum* are still unclear.

Therefore, the purposes of this study were (1) to investigate the effects of high sugar diets and knockdown of *TPS* and *TRE* expressions on the body trehalose and glucose contents of red and green *A. pisum*, (2) to determine the stylet activity thus feeding behavior of these *A. pisum* after the treatments of the high sugar diets and the *TPS* and *TRE* expression knockdown, and (3) to analyse the relationships between *A. pisum* feeding behavior and its physiological trehalose and glucose contents. The results help to provide a theoretical basis for further development of biological agents targeting the feeding behaviors against *A. pisum*.

## Result

### Effect of RNAi and high sugar diets on *ApTPS *and *ApTRE* gene expression

**Figure 1:**
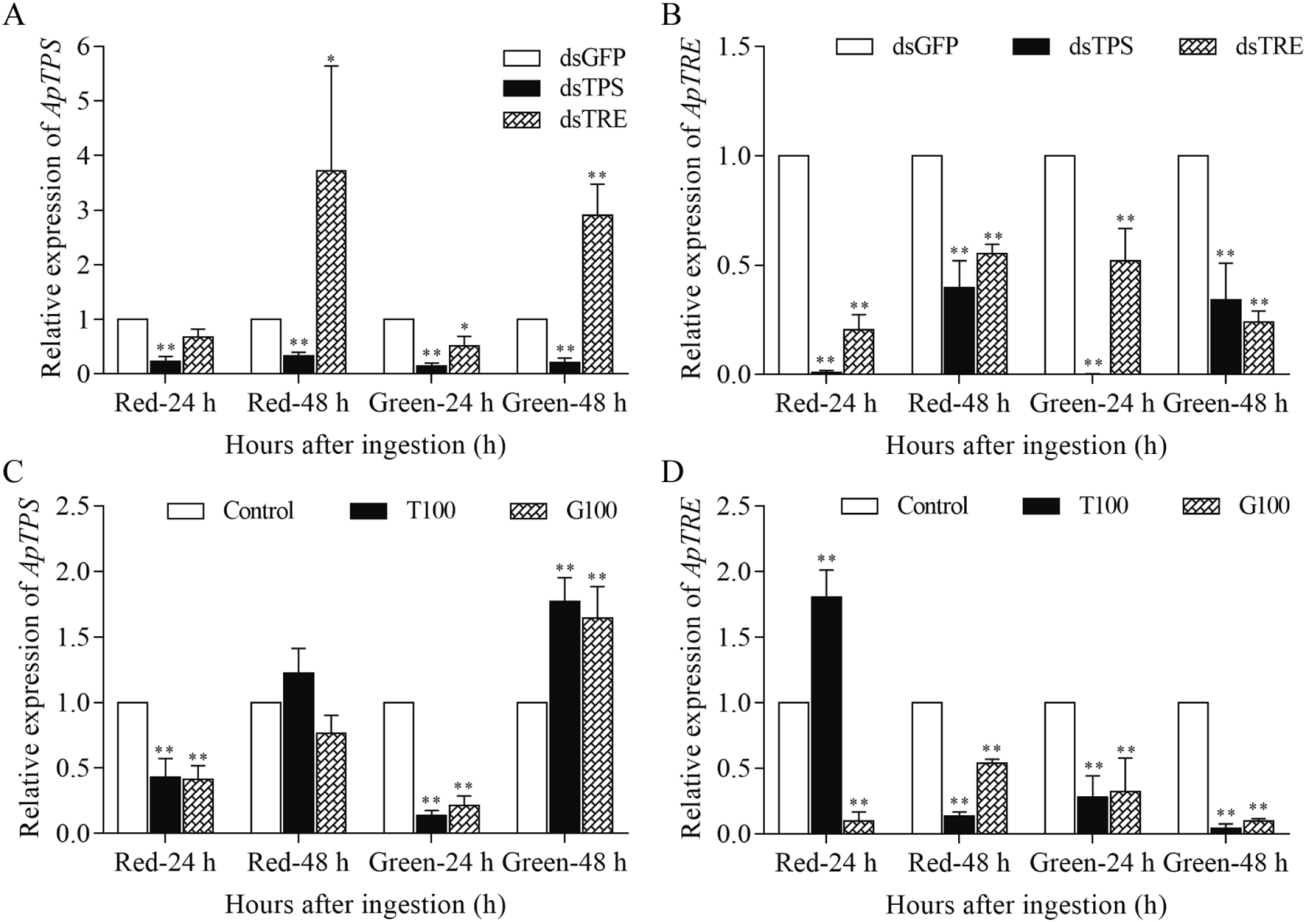
The expression levels of *ApTPS* (**A** and **C**) and *ApTRE* (**B** and **D**). The gene expression level was represented relative to those of normal diet or dsGFP-treated *A. pisum* as fold change and presented as Means ± SEM of three replicates. dsGFP: *A. pisum* treated with RNAi of *GFP*; dsTPS: *A. pisum* treated with RNAi of *TPS*; dsTRE: *A. pisum* treated with RNAi of *TRE*; T100: *A. pisum* treated with high trehalose diet; G100: *A. pisum* treated with high glucose diet; Control: *A. pisum* fed with normal diet. All data were analyzed using Student’s *t*-test. The asterisk indicates significant differences between treatment and control (*P < 0.05, **P < 0.01). Edited in GraphPad Prism version 7.00 (https://www.graphpad.com/scientific-software/prism/).

The *ApTPS* expression was significantly decreased for both red and green biotypes at 24 h and 48 h after the ds*TPS* RNAi treatment (Fig. 1A), and was decreased at 24 h but increased significantly at 48 h after the ds*TRE* RNAi treatment by more than 2 folds in both biotypes compared with that in ds*GPF*-treated *A. pisum* (Fig. 1A). However, the *ApTRE* expression was downregulated by both ds*TPS*- and ds*TRE*-treatment relative to that in the ds*GFP*-treated *A. pisum* (Fig. 1B). Notably, compared with ds*GFP*-treated *A. pisum*, the survival rate of the red biotype was significantly decreased by the RNAi treatments (Fig. S1A) but the survival rate of the green biotype was significantly decreased by RNAi of *ApTRE* (Fig. S1B).

The *ApTPS* expression was downregulated at 24 h and upregulated at 48 h on fresh leave after the high trehalose diet treatment for both biotypes (Fig. 1C). After the high glucose diet treatment, the *ApTPS* expression was upregulated only at 48 h on fresh leave for the green biotype (Fig. 1C). The *ApTRE* expression was downregulated by the high sugar diets in most cases (Fig. 1D), apart from in the high trehalose-treated red biotype where the *ApTRE* expression was upregulated at 24 h on fresh leave after the treatment. The high trehalose diet significantly decreased the survival rate of *A. pisum* compared with that of *A. pisum* on the normal diet (Fig. S1). In addition, the reproduction (the total number of the offspring) was significantly decreased by the *dsTPS* and *dsTRE* treatments and by the high sugar diets (Fig. S2). The expression of *ApTPS* and *ApTRE*, survival and reproduction had a similar trend between red and green biotypes.

### Effect of RNAi and high sugar diets on trehalose and glucose contents

**Figure 2:**
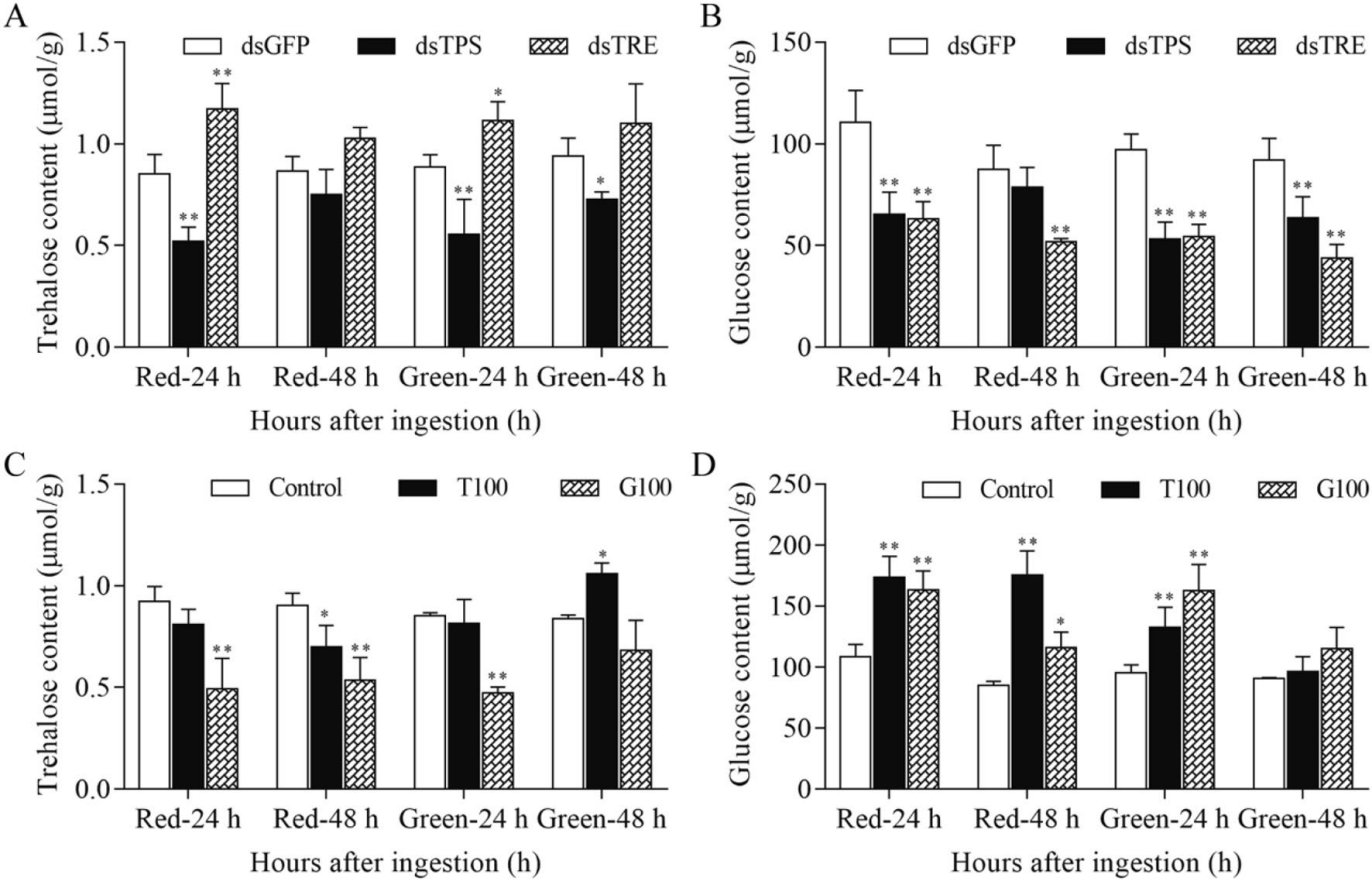
The physiological content of trehalose (**A** and **C**) and glucose (**B** and **D**) in red and green biotypes of *A. pisum*. The contents of trehalose and glucose were presented as Means ± SEM of three replicates. dsGFP: *A. pisum* treated with RNAi of *GFP*, dsTPS: *A. pisum* treated with RNAi of *TPS*, dsTRE: *A. pisum* treated with RNAi of *TRE*, T100: *A. pisum* treated with high trehalose diet, G100: *A. pisum* treated with high glucose diet, Control: *A. pisum* fed with normal diet. All data were analyzed using Student’s *t*-test. The asterisk indicates significant differences between treatment and control (*P < 0.05, **P < 0.01). Edited in GraphPad Prism version 7.00 (https://www.graphpad.com/scientific-software/prism/).

The trehalose contents were decreased in the ds*TPS*-treated *A. pisum* but increased in the ds*TRE*-treated *A. pisum* in all cases compared with those in ds*GFP*-treated *A. pisum* (Fig. 2A). The glucose contents were decreased in both ds*TPS*-treated and ds*TRE*-treated *A. pisum* at both time points (24 h and 48 h) for both red and green biotypes (Fig. 2B). The trehalose contents were decreased in the red biotype but increased in the green biotype by the high trehalose diet at 48 h (Fig. 2C). It was decreased by the high glucose diet at both time points (Fig. 2C). However, both high sugar diets increased the glucose contents (Fig. 2D). In addition, the content of trehalose and glucose had a similar trend between red and green biotypes.

### Effect of RNAi and high sugar diets on feeding behavior

**Figure 3:**
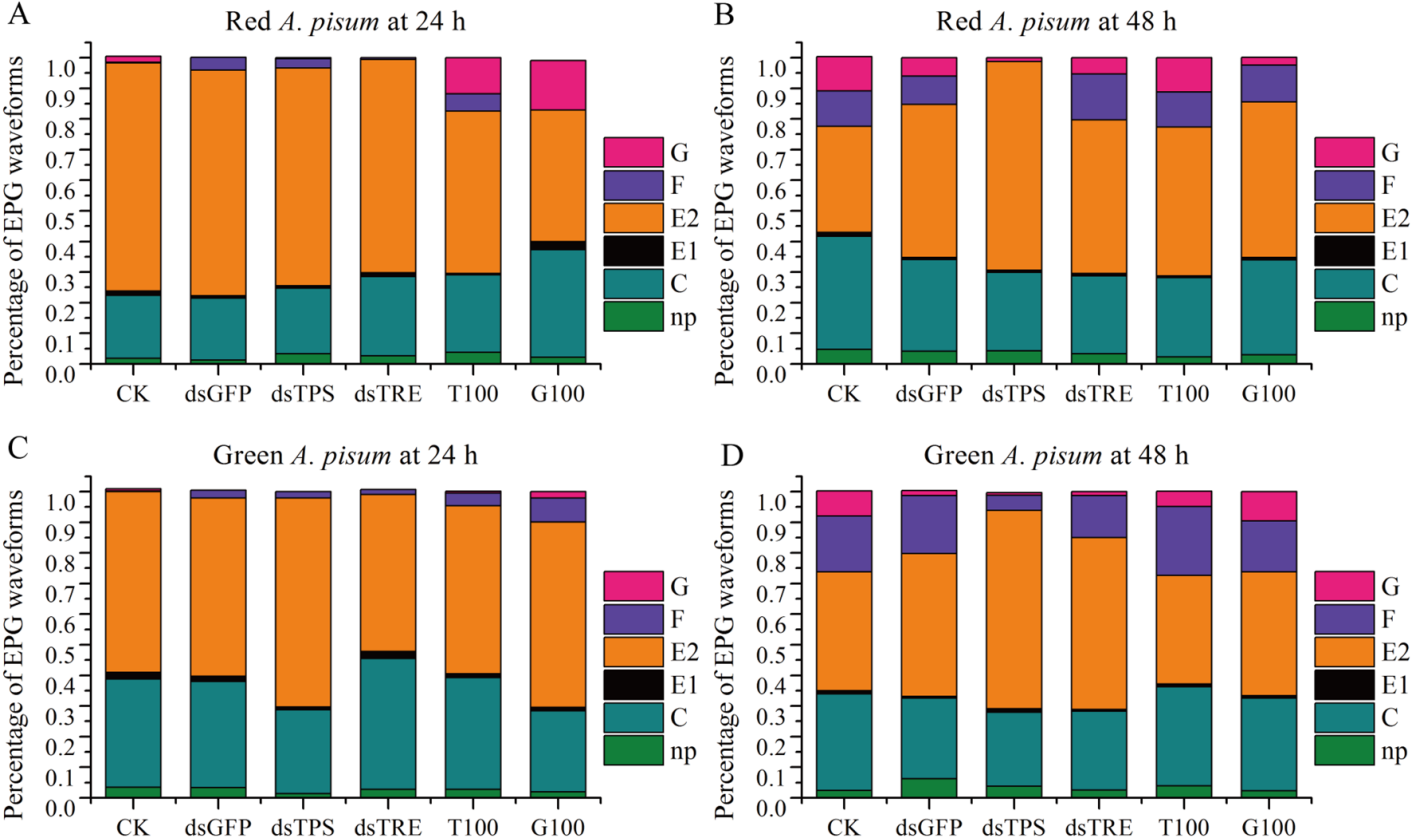
Mean percentage of the EPG waveforms during 8 h EPG recording. The percentages of EPG waveforms in red *A. pisum* biotype at 24 h and 48 h were resented in (**A** and **B**), repectively. The percentages of EPG waveforms in green *A. pisum* at 24 h and 48 h were resented in (**C** and **D**), respectively. dsGFP: *A. pisum* treated with RNAi of *GFP*, dsTPS: *A. pisum* treated with RNAi of *TPS*, dsTRE: *A. pisum* treated with RNAi of *TRE*, T100: *A. pisum* treated with high trehalose diet, G100: *A. pisum* treated with high glucose diet, CK: *A. pisum* fed with normal diet. The waveform for xylem ingestion (G), waveform for derailed stylet mechanics (F), waveform for phloem ingestion (E2), waveform for phloem salivation (E1), waveform for intercellular apoplastic stylet pathway (C) and non-probing (np) are present in different colours. Edited in Origin version 8.5 (https://www.originlab.com/).

Figure 3 shows the feeding activities recorded as EPG waveforms when the *A. pisum* probes into plants and presented as the percentage of each EPG waveform. An overview of the representative EPG waveforms of treated and control *A. pisum* on both time points is shown in Figs. S3, S4, S5 and S6. At 24 h on fresh leaves after the treatments, no significant change in any EPG waveform was found in the ds*TPS*-treated and ds*TRE*-treated red biotype *A. pisum* compared with the ds*GFP*-treated *A. pisum*, which was not different from those of the untreated *A. pisum* (CK) (Fig. 3A; Table S2A). In the green biotype *A. pisum*, the percentage of E2 waveform was increased by the ds*TPS*-treatment and decreased by the ds*TRE*-treatment (Fig. 3C; Table S2C). The number of each waveform was not different in both ds*TPS*-treated and ds*TRE-*treated groups relative to that of the ds*GFP*-treated group (Table S2A and S2C). The high sugar diets decreased and increased the percentage of E2 waveform of the red and green biotype *A. pisum*, respectively. The high sugar diets also increased the percentage of G waveform of the red biotype *A. pisum* (Fig. 3A; Table S2E) and the percentage of F waveform of the green biotype *A. pisum* (Fig. 3C; Table S2G). Interestingly, the high trehalose diet significantly reduced the number of E2 waveform and the high glucose diet significantly elevated the number of C, E1 and pd waveforms in the red *A. pisum* (Table S2E).

At 48 h on fresh leaves after the ds*TPS*-treatments, the percentage of E2 waveform was increased, and the percentage of F and G waveforms were decreased compared with those of the CK *A. pisum* (Fig. 3B and D; Table S2B and S2D), but the ds*TRE*-treatment did not affect the percentage of any waveform compared with the CK group (Fig. 3B and D; Table S2B and S2D). Notably, the number of F waveform was significantly reduced in the ds*TPS*-treated *A. pisum* compared with ds*GFP*-treated *A. pisum* (Table S2B and S2D). The high sugar diets had little effect on the EPG waveforms (Fig. 3B and D; Table S2F and S2H) of both the red and green biotype *A. pisum*. The high glucose diet significantly reduced the number of G waveform in the red biotype *A. pisum* (Table S2F). In addition, the feeding behaviors had a similar trend between red and green biotype *A. pisum*. Notably, the EPG waveforms were of huge difference between the treatment groups at 48 h (Fig. 3B and D).

### Relationships between feeding behavior and physiological sugar contents

**Figure 4:**
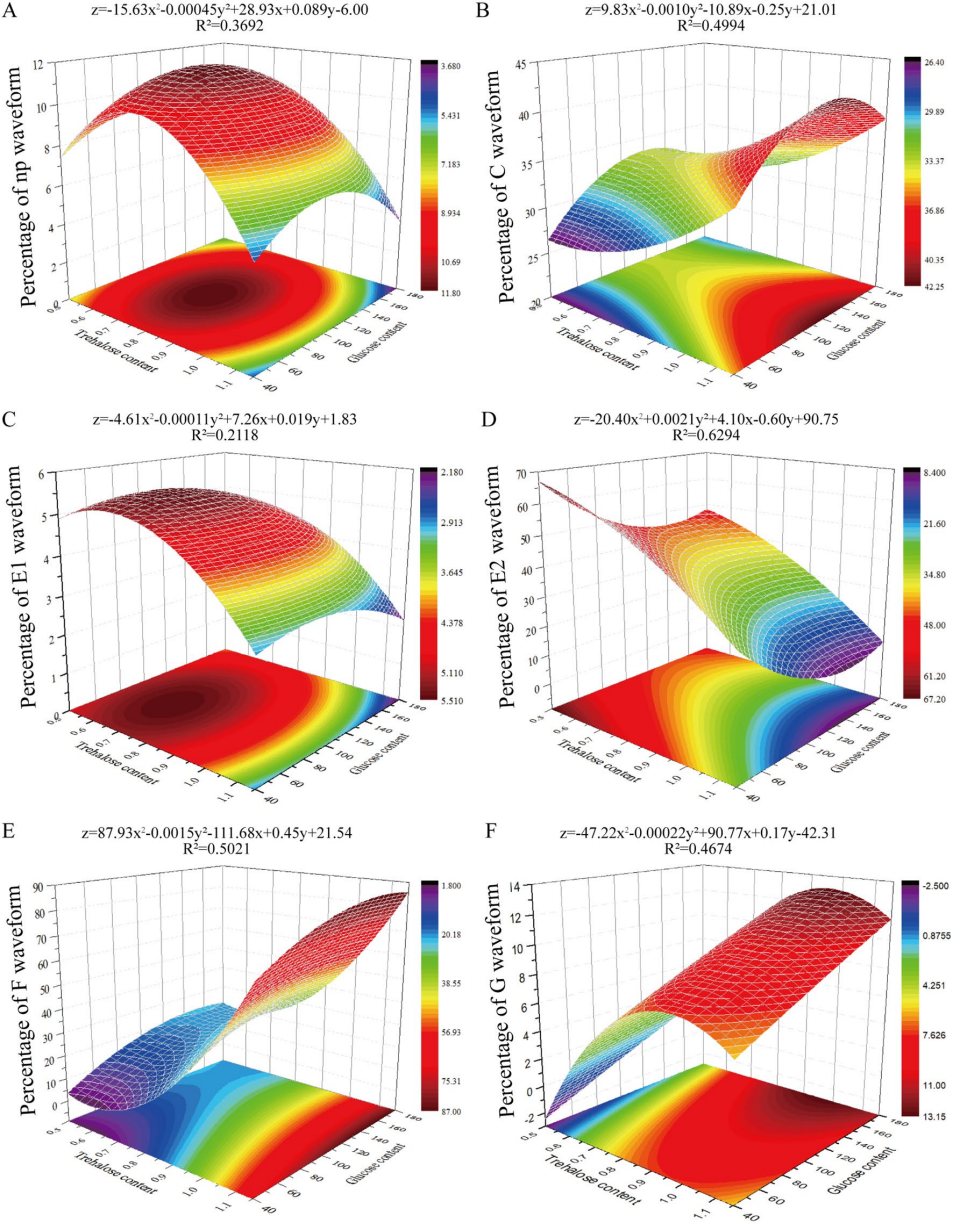
Relationship between the percentages of EPG waveforms and the sugar contents of *A. pisum*. The percentage of each waveform is the mean of three measurements at 48 h after each treatment (dsTPS, dsTRE, dsGFP, T100, G100 and normal diet). Z-axis: the percentage of EPG waveforms; X-axis: the trehalose content; Y-axis: the glucose content. The contents of trehalose and glucose were used in the curve fitting as $z = a_{1} x^{2} + a_{2} y^{2} + a_{3} x + a_{4} y + b$, where z is normalized percentage of EPG waveforms by arcsine square-root transformation; x is trehalose contents; y is glucose contents; a1, a2, a3, and a4 are coefficients; b is constant. The 5 parameters were fitted by 12 points data of trehalose contents, glucose contents, and EPG waveforms percentages at 48 h. The np waveform (**A**), C waveform (**B**), E1 waveform (**C**), E2 waveform (**D**), F waveform (**E**), and G waveform (**F**) are presented. The percentages of EPG waveforms are presented as the Z-plane and bottom contour in the coulor scale from dark red to blue for the highest and lowest percentages. Edited in Origin version 8.5 (https://www.originlab.com/).

To illustrate the relationships of physiological sugar levels on the feeding behavior, the sugar (trehalose and glucose) contents and the percentages of EPG waveforms obtained at 48 h before and after the treatments were assayed using curve fitting $z = a_{1} x^{2} + a_{2} y^{2} + a_{3} x + a_{4} y + b$ where z is the arcsine square-root transformation of the percentage of EPG waveform, x is the trehalose contents, and y is the glucose contents. The fitting plane in Fig. 4A shows that the percentage of np waveform gradually increases with the elevation of the sugar contents, reaching the highest percentage (11.80) where the trehalose content is 0.77 µmol/g and the glucose content is 100.20 µmol/g (${\text{z}} = - 15.63{\text{x}}^{2} - 0.00045{\text{y}}^{2} + 28.93{\text{x}} + 0.089{\text{y}} - 6.00$) (Fig. 4A). The R^2^ of the fitting is 0.3692, indicating a moderate correlation. The percentage of C waveform increases with the increasing of trehalose content, and as the glucose content raises it first increases and then decreases. It has the highest percentage (42.21) at 121.27 µmol/g of the glucose content and a high trehalose content (1.15 µmol/g) (${\text{z}} = 9.83{\text{x}}^{2} - 0.0010{\text{y}}^{2} - 10.89{\text{x}} - 0.25{\text{y}} + 21.01$) (Fig. 4B) and a high correlation with the sugar contents (*R*^2^ = 0.4994). The percentage of E1 waveform shows a similar trend as the percentage of np waveform with the highest percentage (5.50) at the point where the trehalose content is 0.68 µmol/g and the glucose content is 84.82 µmol/g (${\text{z}} = - 4.61{\text{x}}^{2} - 0.00011{\text{y}}^{2} + 7.26{\text{x}} + 0.019{\text{y}} + 1.83$), and has a similar weak correlation (R^2^ = 0.2118) (Fig. 4C). The percentage of E2 waveform gradually decreases with the trehalose content elevation, and decreases first and then increases with the glucose content elevation, reaching the lowest point (8.46) at 140.8 µmol/g of glucose content and 1.15 µmol/g of trehalose content, and the highest percentage (67.10) at 0.5 µmol/g of trehalose content and 40 µmol/g of glucose content (${\text{z}} = - 20.40{\text{x}}^{2} + + 0.0021{\text{y}}^{2} + 4.10{\text{x}} - 0.60{\text{y}} + 90.75$) (Fig. 4D). The high *R*^2^ of 0.6294 suggests that the E2 waveform has a good correlation with the sugar contents. The percentage of F waveform first decreases and then increases as the trehalose content increases to the lowest point (1.82) at 0.58 µmol/g of trehalose, and as the glucose content raises it increases first and then decreases and reaches the highest percentage (86.85) at 153.41 µmol/g of glucose content (${\text{z}} = 87.93{\text{x}}^{2} - 0.0015{\text{y}}^{2} - 111.68{\text{x}} + 0.45{\text{y}} + 21.54$) (Fig. 4E). Its correlation with the sugar content is high (*R*^2^ = 0.5021). Finally, the percentage of F waveform increases first and then decreases as the trehalose content raises and has the highest point (13.12) at 0.955 µmol/g of the trehalose content and 180 µmol/g of the glucose content (${\text{z}} = - 47.22{\text{x}}^{2} - 0.00022{\text{y}}^{2} + 90.77{\text{x}} + 0.17{\text{y}} - 42.31$) (Fig. 4F) with a high correlation (*R*^2^ = 0.4674) with the sugar contents.

These analyses clearly illustrate the aphid feeding behaviors under different body sugar contents. Thus, the low physiological sugar levels of the aphids increase E1 and E2 waveforms but decrease np, C, F and G waveforms. Interestingly, the high physiological sugar levels reduce the percentage of np, E1 and E2 waveforms but elevate the percentage of C, F and G waveforms.

## Discussion

Sugar metabolism plays a critical role in the adaptation of aphids to various environmental conditions and in the regulation of survival, reproduction and feeding behavior. Our results showed that the feeding on a high trehalose diet did not increase *A. pisum* physiological trehalose content, but increased glucose content (Fig. 2C and D). The trehalose contents were decreased by the high glucose diet (Fig. 2C) and the glucose contents were increased by both high sugar diets (Fig. 2D), suggesting that the glucose level is readily regulated in *A. pisum*. The aphids may utilize glucose at a very lower level, so are sensitive to the change of the glucose level, while trehalose is stored as an energy resource. It is possible that, when the trehalose content is very high, it would be hydrolyzed to produce glucose. It was reported that gluconeogenesis contributed greatly to sugar contents in insects maintained on a low carbohydrate diet, but on a high carbohydrate diet, the sugar contents was derived mainly from dietary carbohydrate, whereas the generation of amino acids was regulated post-ingestively^21,28^.

The RNAi of *ApTPS* decreased the trehalose content of *A. pisum* as in *B. minax* and *L. decemlineata*^18,25^. However, the trehalose contents were increased in the ds*TRE*-treated *A. pisum* (Fig. 2A). This is contradictory to the report in *S. exigua* larvae^26^. Thus, the effects of RNAi of *TRE* on the glucose content may be different in different insect species. The glucose contents were decreased in the ds*TPS*- and ds*TRE*-treated *A. pisum* at both time points (24 h and 48 h) for both red and green biotypes (Fig. 2B), further confirming the sensitive regulation of the glucose level in *A. pisum*.

It was also observed in this study that the high trehalose diets not only affect the survival rate but also reduced the reproduction of red and green *A. pisum* (Figs. S1 and S2) in agreement with previous studies^18,23,26^ in *L. decemlineata*^18^, *Drosophila melanogaster*^23^ and *Harmonia axyridis*^29^. Knockdown of adipokinetic hormone receptor gene reduced egg number produced and the fecundity of *N. lugens* by decreasing the trehalose contents in hemolymph^30^. In this study, the RNAi of *ApTPS* and *ApTRE* reduced the survival rate of the red biotype of *A. pisum* (Fig. S1). These results are consistent that knockdown of *TPS* significantly lowed survival rate of *N. lugens*^15,24^, *Tribolium castaneum*^31^ and *B. minax*^25^, and that *S. exigua*^26^, *L. decemlineata*^18^ and *T. castaneum*^32^, these results are consistent that RNAi of *TRE* decreased survival rate of *S. exigua*^26^, *L. decemlineata*^18^ and *T. castaneum*^32^. These results indicate that the maintaining of trehalose metabolic balance is important for insect life cycle.

The high sugar diets and the RNAi of *ApTRE* did not significantly change the percentage of each EPG waveform. The difference in the percentage of EPG waveforms between treatment and control groups was observed only at 48 h (Fig. 3; Table S2). RNAi of *ApTPS* increased the percentage of E2 waveform and decreased the percentage of F and G waveforms. Overall, the *A. pisum* spent more time on E2 waveform (phloem-feeding) (Fig. 3). This is consistent with the phloem-feeding activity of *A. pisum* for nutrients^33^. Notably, the number of F waveform was significantly reduced in the RNAi of *ApTPS* (Table S2B and S2D), suggesting that RNAi of *ApTPS* promotes the bundle formation^4^, probably due to the differences in salivary components^34^. However, these results are opposite to the increase of the number of F waveform in the neuropeptide F gene knockdown aphids^35^, aposymbiotic aphids^4^ and feeding on resistant plant aphids^36^.

Insect feeding behavior is influenced primarily by two factors: metabolic needs and satiety^37–39^. In *Drosophila*, metabolizable sugars food choice correlates with low hemolymph sugar levels^37^. *Tenodera sinensis* mantises directed their attention toward real and simulated prey less often as they sated^39^. In this study, the physiological sugar levels was changed but the satiety was not changed by the free diet, suggesting *A. pisum* feed behavior is relies on their metabolic needs. It was reported that high trehalose diets negatively affected food intake of *L. decemlineata* and *S. exigua*^18,26^. High sucrose diets decreased the consumption rate of *A. pisum*, and a low sucrose diet increased food ingestion of *Ceratitis capitata* female by 35% compared with the control^22,40^. Trehalose and glucose are the two main sugars in the insect body and they plays important role in food-choice behaviour^20^. The measurements of trehalose and glucose contents and the feeding behaviors of *A. pisum* treated with RNAi and high sugar diets provided a unique opportunity to analyse the relationships of the physiological sugar levels with the feeding behaviors of *A. pisum*. The curve fitting analysis showed that the aphids with low trehalose and glucose contents had a low feeding activity during non-probing phase (np waveform; Fig. 4A), probing phase (C waveform; Fig. 4B), stylet work phase (F waveform; Fig. 4E), and xylem ingestion phase (G waveform; Fig. 4F) but had an increased feeding activity during phloem phase (E1 and E2 waveforms; Fig. 4C and D, respectively). However, the high trehalose and high glucose contents increased the aphid feeding activity during probing phase (Fig. 4B), stylet work phase (Fig. 4E), and xylem ingestion phase (Fig. 4F) but decreased the aphid feeding activity during non-probing phase (Fig. 4A), phloem phase (Fig. 4C and D). These data indicate that the level of trehalose and glucose is an important factor that influnces the feeding behavior in *A. pisum*. The increase of phloem-feeding time under the low physiological sugar levels is a sign that the *A. pisum* needs more carbohydrates to maintain its homeostasis. However, the high physiological sugar levels increase aphid phloem-feeding for more water to balance the body’s high physiological sugar level. Interestingly, the high physiological sugar levels increased the activity of probing phase (Fig. 4B) and stylet work phase (Fig. 4E), indicating that *A. pisum* spent more time feeding in the cell walls, intercellular spaces of vascular tissue, and the mesophyll as when *A. pisum* aphids feed on resistant plants^4,34,36,41^.

In conclusion, this study shows that RNAi of *ApTPS* and high sugar diets can affect the trehalose and/or glucose content in the body of *A. pisum*. This allows to analyse the relationships between sugar contents and feeding behaviors under physiological conditions. It provides strong evidence that the feeding behavior of *A. pisum* is influenced by the level of trehalose and glucose in the body. This is the first report using the EPG technique to study the link of *A. pisum* physiological sugar level and feeding behavior. Future research is now required to validate the mechanism of physiological sugar level regulated feeding behaviour.

## Materials and methods

### Plant and culture conditions

The study was carried out under artificial climate incubator at 20 ± 1 ℃, 70 ± 10% relative humidity, with a photoperiod of 16 h L: 8 h D. Seeds of *Vicia faba* ’Lincan-9’ were provided by the NingXia Academy of Agricultural and Forestry Sciences. All plants were cultured in 9 cm diameter pots. When seedlings grew to the 4–5 leaf stage for use in the experiments. The experiment did not involve any endangered or protected species. All experimental research on the above mentioned plants, complies with relevant institutional, national, and international guidelines and legislation.

### Insect and culture conditions

Clones of red and green morphs of *A. pisum* were established from single virginiparous females. Samples were collected in 2017 from same Alfalfa plant *Medicago sativa* in field, Lanzhou, China, and reared on the fava bean *Vicia faba* in the laboratory. All plants and *A. pisum* cultures were reared in an artificial climate incubator at 20 ± 1 ℃, 70 ± 10% relative humidity, with a photoperiod of 16 h L: 8 h D. Mature *A. pisum* were put on a fava bean leaf for 12 h and the resulting neonate nymphs, 0–12 h old, were used for experiments throughout this study.

### RNA isolation and first-strand cDNA synthesis

Total RNA was isolated using TRizol reagent (BBI Life Sciences, Shanghai, China) following the manufacturer’s instructions. The total quantity of extracted RNA was assessed using a micro-volume UV spectrophotometer (Quawell Q5000, Quawell, USA). The RNA integrity was confirmed further by 1% formaldehyde agarose gel electrophoresis. Total RNA was dissolved in 50 µL DEPC-water and stored at − 80℃. The first-strand cDNA was synthesized using a First-Strand cDNA Synthesis kit (BioTeke, Beijing, China) and stored at − 20℃ for subsequent experiments.

### Cloning of TPS and TRE cDNAs

The primer sets, TPS-F/R of *ApTPS* and TRE-F/R of *ApTRE*, were designed using the primer software Primer 5.0 (Premier Biosoft, Palo Alto, CA, USA) based on the TPS gene sequence (GENBANK accession: XM_001943581.5) and the TRE gene sequence (GENBANK accession: XM_003245847.4) of *A. pisum*. The primers of the green fluorescent protein gene (*GFP*, pET28a-EGFP, Miaolingbio, Wuhan, China) were referenced from Yang et al.^15^. These primers are listed in Table S1. The components of the PCR reaction mixture included 1.0 µL of the template (1 ng/µL), 12.5 µL 2 × Power Tap PCR MasterMix (BioTeke, Beijing, China), 1.0 µL of each primer (10 µmol/µL), and 9.5 µL Rnase-free H_2_O concentration for a final volume of 20 µL. The PCR reaction conditions were pre-denatured at 95 ℃ for 5 min, followed by 35 cycles of 95 ℃/45 s for denature 55 ℃/45 s for annealing and 72 ℃/1 min for extension, and then 10 min at 72 ℃ for a final extension. PCR products were subjected to 1.0% agarose gel electrophoresis and purified by DNA gel extraction kit (BioTeke, Beijing, China). The purified DNA was ligated into the pMD18-T vector (TaKaRa, Dalian, China) and sequenced by Tsing Ke Biological Technology (Tsing Ke Biological Technology, Beijing, China) using the dideoxynucleotide method. The lengths of the resulting *ApTPS*, *ApTRE*, and *GFP* genes were 421 bp, 416 bp, and 688 bp, respectively.

### dsRNA synthesis

Three pairs of primers (dsTPS-F/R, dsTRE-F/R and dsGFP-F/R), with the T7 RNA promoter sequence flanking the 5’-end of each gene, were designed and synthesized (Table S1), and used to make the templates for in vitro dsRNA transcription via PCR. The dsRNAs were synthesized using the TranscriptAid T7 High Yield Transcription Kit (Thermo Scientific, Wilmington, DE, USA) according to the manufacturer’s protocol^42^. The size of the dsRNA products was confirmed by electrophoresis on a 1.5% agarose gel and the concentration was assessed using a micro-volume UV spectrophotometer.

### dsRNA and high sugars diet treatments

The artificial diet bioassay was performed according to the following procedure^43^. A liquid artificial diet was prepared as described previously^44,45^, filtered through a 2 µm membrane, dispensed in 1.0 mL aliquots, and stored at -20℃ before assays. The testing diets were prepared by adding either each of dsRNA (ds*TPS*, ds*TRE* and ds*GFP*) or each of sugar (trehalose and glucose) to the 1.0 mL artificial diet for a final concentration of 400 ng/µL (dsRNA) and 100 µg/mL (sugar). The diet containing nuclease-free water was used as control of the high sugar diet treatments and diet containing ds*GFP* was used as control of the RNAi treatments. There was a total of 6 treatments including two controls for either red or green *A. pisum*.

Glass vials (2.5 cm in diameter) were sterilized for the aphid artificial double-membrane feeding assay and one opening was completely sealed with parafilm. Seventy microliters of the testing diet were placed on the parafilm and covered with parafilm. So the testing diet was sandwiched between two layers of the parafilm membrane at one opening of the glass vials^45^. The control group was fed with only the artificial diet without dsRNA or sugars.

Fifteen 3-day-old *A. pisum* were introduced into one vial, and the vial was closed with a piece of sterilized gauze as one of bioassays. The artificial diet was replaced every other day to prevent dsRNA degradation. After 4 days, all surviving *A. pisum* were transferred to fresh bean leaf discs.

### Quantification of gene expression levels after RNAi and high sugar diet treatments

Seven *A. pisum* were collected from fresh bean leaf discs at 24 h and 48 h after the 4-day treatment with the testing diet containing each of dsRNAs. *A. pisum* were immediately frozen in liquid nitrogen and three replicates were carried for each treatment. Total RNA was isolated from the seven pooled whole *A. pisum* bodies. The first-strand cDNA was synthesized from total RNA using a First-Strand cDNA Synthesis kit (BioTeke, Beijing, China). The RT-qPCR analysis was carried out in 96-well 0.1-mL block plates using a QuantStudio™ 5 system (Thermo Scientific, Wilmington, DE, USA). Each reaction contained 1.0 µL of the cDNA template, 10.0 µL 2 × Plus SYBR real-time PCR mixture (BioTeke, Beijing, China), 0.5 µL of each primer (10 µmol/µL), 8 µL EDPC-ddH_2_O, and 0.5 µL 50 × ROX Reference Dye concentration for a final volume of 20 µL. The RT-qPCR reaction conditions were pre-denatured at 94 ℃ for 2 min, by 40 cycles of 94 ℃/15 s, and 55–62 ℃/30 s for annealing. After each reaction, a melting curve analysis (denatured at 95 ℃ for 15 s, annealed at 60℃ for 1 min, and denatured at 95 ℃ for 15 s) was conducted to ensure consistency and specificity of the amplified product. Three biological replicates and three technical replicates were set for each treatment in the RT-qPCR analysis. Quantification of the transcript level was conducted according to the $2^{ - \Delta \Delta Ct}$ method^46^, and the ribosomal protein L27 gene (*rpL27*) was used as a reference gene^47^.

### Trehalose and glucose content assays after RNAi and high sugar diet treatments

Ten *A. pisum* were collected from fresh bean leaf discs at 24 h and 48 h after the 4-day treatment with the testing diet containing each of sugars. *A. pisum* were immediately frozen in liquid nitrogen and three replicates were carried for each treatment. The trehalose content assay was conducted according to the method described by Yang et al.^15^. Briefly, ten whole *A. pisum* bodies were ground in phosphate-buffered saline (PBS: 130 mM NaCl; 7 mM Na_2_HPO_4_·2H_2_O; 3 mM NaH_2_PO_4_·2H_2_O; pH 7.0), and then a 25 µL of tissue was taken and uniformly mixed with 25 µL of 1% sulfuric acid. The mixture was incubated at 90 ℃ for 10 min and placed in ice for 3 min, and then 25 µL of 30% potassium hydroxide solution was added into the sample and mixed uniformly. The resultant mixture was incubated at 90 ℃ for 10 min and then in ice for 3 min. Finally, 500 µL of 0.2% anthrone reagent was added to the sample and incubated at 90 ℃ for 10 min and then in ice for 3 min. The trehalose content was assayed by measuring the absorbance of the final reaction mixture at 630 nm. The glucose content was determined using the glucose assay kit (Solarbio Biochemical Assay Division, Beijing, China) according to the manufacturer’s protocols.

### Evaluation of *A. pisum* feeding behavior

The probing behavior was evaluated with the electrical penetration graph (EPG) using an 8-channel DC-EPG device (Wageningen University, the Netherlands). Eight plants were placed in a faraday cage, and wingless *A. pisum* were placed on the abaxial side of the second fully expanded leaf from the top. Before exposure *A. pisum* to the plant, a 6 to 8 cm long gold wire (diameter 18 µm) was conductively glued (water-based silver glue) to *A. pisum* dorsum as the recording electrode. The other end of the gold wire was attached to a 3 cm long copper wire (diameter 0.2 mm) which was connected to the first head stage on the DC-EPG amplifier with the setting of 1 Giga-Ohm input resistance and 50 × gain. The reference electrode was inserted into the soil and connected to the plant voltage output of the DC-EPG device. *A. pisum* from each treatment was randomly distributed during recording. For each treatment, only the *A. pisum* that showed activities in an 8 h recording period were considered as valid replicates.

The EPG signal was recorded by the Stylet + d software and the EPG waveforms were recognized and labeled using Stylet + av01.30 software (EPG Systems, Wageningen, Netherlands). The EPG parameters were calculated for each *A. pisum* treatment using the Excel workbook for automatic parameter calculation of EPG data 4.4.3^48^ and then the means and standard errors of the mean (SEM) were calculated for each treatment at 24 h and 48 h on fresh bean leaf discs after 4-day treatments.

### Survival and reproduction assays

*A. pisum* were reared on fresh bean leaf discs after the treatments in an artificial climate incubator at 20 ± 1 ℃, 70 ± 10% relative humidity, with a photoperiod of 16 h L: 8 h D. Survival and reproduction assays were conducted for the control and treated *A. pisum*. The daily numbers of adult *A. pisum* deaths and newborn nymphs per adult *A. pisum* were recorded until they no longer produced nymphs, once per day starting from the first day after the treatments.

### Curve fittings

The relationships between the normalized percentage of EPG waveforms by arcsine square-root transformation under each treatment (z) and the corresponding physiological trehalose content (x) and glucose content (y) were then analyzed as $z = a_{1} x^{2} + a_{2} y^{2} + a_{3} x + a_{4} y + b$ by curve fitting with the software 1stOpt 15.0 (7D-Soft High Technology lnc, China), where a_1_, a_2_, a_3_ and a_4_: coefficients; b: constant. The 5 parameters were fitted by 12 points data of trehalose content, glucose content, and EPG waveforms percentage at 48 h. The 48 h trehalose and glucose content data of the *A. pisum* obtained in Materials & Methods 2.7 and the percentage of EPG waveforms data of the *A. pisum* obtained in Materials & Methods 2.8.

### Statistical analysis

All statistical analyses were performed using 1stOpt 15.0 and SPSS 19.0, and Origin 8.5 and GraphPad Prism 7.00 software were used to produce charts. The RT-qPCR and sugar data were analyzed by Student’s *t*-test. The survival data were subjected to a Kaplan–Meier survival log-rank analysis (Fig. S1)^35^. The EPG data (Table S2) and the total reproduction data (Fig. S2) were analyzed using one-way analysis of variance (ANOVA) followed by the Tukey’s post hoc test. A *p*-value < 0.05 was considered statistically significant.

## Supplementary Information

Supplementary Information.
