# Supplementary material for: Trehalose and glucose levels regulate feeding behavior of the phloem-feeding insect, the pea aphid Acyrthosiphon pisum Harris
Source: Sci Rep. 2021 Aug 5;11:15864. doi: 10.1038/s41598-021-95390-z (PMC8342477; doi:10.1038/s41598-021-95390-z)
Supplement: Supplementary file 1 — Supplementary Information. [file 41598_2021_95390_MOESM1_ESM.docx]

Trehalose and glucose levels regulate feeding behavior of the phloem-feeding insect, the pea aphid *Acyrthosiphon pisum* Harris

Guang Wang^1,2^, Jing-Jiang Zhou^1,3^, Yan Li^1,2^, Yuping Gou^1,2^, Peter Quandahor^1,2^ & Changzhong Liu^1,2,*^

^1^ College of Plant Protection, Gansu Agricultural University, Lanzhou, 730070, China

^2^ Biocontrol Engineering Laboratory of Crop Diseases and Pests of Gansu Province, Lanzhou, 730070, China

^3^ State Key Laboratory of Green Pesticide and Agricultural Bioengineering, Ministry of Education, Guizhou University, Huaxi District, Guiyang 550025, China

* Corresponding author: Changzhong Liu, Email: [liuchzh@gsau.edu.cn](mailto:liuchzh@gsau.edu.cn)

**Supplementary Materials:**

**1. Tables**

**Table S1.** The primers of DNA amplification, dsRNA synthesis and RT-qPCR.

| Genes | Primer set | Primers sequence (5’-3’) | Product Size (bp) |  |
| --- | --- | --- | --- | --- |
| *TPS* | TPS-F | GGATGCAACCTACCACCCAA | 421 |  |
|  | TPS-R | CCCGGTAGTGATACGTCAGC |  |  |
| *TRE* | TRE-F | GGCGTGGTTTGACTGGGATA | 416 |  |
|  | TRE-R | CAACCAAAACCTGTCTGCGG |  |  |
| *GFP* | GFP-F | AAGGGCGAGGAGCTGTTCACCG | 688 |  |
|  | GFP-R | CAGCAGGACCATGTGATCGCGC |  |  |
| *dsTPS* | dsTPS-F | TAATACGACTCACTATAGGGGGATGCAACCTACCACCCAA | 421 |  |
|  | dsTPS-R | TAATACGACTCACTATAGGGCCCGGTAGTGATACGTCAGC |  |  |
| *dsTRE* | dsTRE-F | TAATACGACTCACTATAGGGGGCGTGGTTTGACTGGGATA | 416 |  |
|  | dsTRE-R | TAATACGACTCACTATAGGGCAACCAAAACCTGTCTGCGG |  |  |
| *dsGFP* | dsGFP-F | TAATACGACTCACTATAGGGAAGGGCGAGGAGCTGTTCACCG | 688 |  |
|  | dsGFP-R | TAATACGACTCACTATAGG*G*CAGCAGGACCATGTGATCGCGC |  |  |
| *TPS* | qTPS-F | ACCGACCGTGCCAATTTCGTTT | 136 |  |
|  | qTPS-R | CGAAACCAAGGGGGCTGTTTG |  |  |
| *TRE* | qTPS-F | CGCATTGTACGCTGGTCATT | 80 |  |
|  | qTPS-R | AGTTGGACCCTGAGACTCCT |  |  |
| *rpL27* | qTPS-F | CCGAAAAGCTGTCATAATGAAGACC | 231 |  |
|  | qTPS-R | GGTGAAACCTTGTCTACTGTTACATCTTG |  |  |

Note: T7 RNA promoter sequence: TAATACGACTCACTATAGGG

**Table S2.** The EPC parameters of red and green *A. pisum*.

**A.** Means ± SEM of EPG variables for dsGFP, dsTPS and dsTRE treated red *A. pisum* feeding on *Vicia faba* after 24 h, during 8 h.

| Variables | Related to | dsGFP (n = 15) | dsTPS (n = 18) | dsTRE (n = 14) | *P* value |
| --- | --- | --- | --- | --- | --- |
| *Number of events of each waveform, per insect*.* | | | | | |
| np | All tissues | 4.53 ± 1.04 a | 7.00 ± 1.17 a | 8.86 ± 2.53 a | 0.201 |
| C | All tissues | 8.07 ± 1.48 a | 9.22 ± 1.50 a | 13.29 ± 2.93 a | 0.183 |
| E1 | Phloem | 4.20 ± 0.99 a | 3.28 ± 0.72 a | 6.00 ± 1.40 a | 0.177 |
| E2 | Phloem | 3.67 ± 0.89 a | 2.56 ± 0.44 a | 4.71 ± 1.03 a | 0.157 |
| F | Epidermis and mesophyll | 0.20 ± 0.11 a | 0.17 ± 0.09 a | 0.07 ± 0.07 a | 0.618 |
| G | Xylem | 0.00 ± 0.00 a | 0.11 ± 0.08 a | 0.00 ± 0.00 a | 0.195 |
| pd | Epidermis and mesophyll | 85.47 ± 13.59 a | 77.94 ± 13.05 a | 116.43 ± 18.57 a | 0.178 |
| probes | All tissues | 4.40 ± 1.05 a | 7.00 ± 1.17 a | 8.79 ± 2.51 a | 0.188 |
| *Total duration events of each waveform, per insects (min)**.* | | | | | |
| np | Epidermis and mesophyll | 6.16 ± 1.88 a | 16.01 ± 4.27 a | 12.61 ± 3.88 a | 0.154 |
| C | Epidermis and mesophyll | 97.11 ± 13.15 a | 102.63 ± 15.70 a | 124.05 ± 17.78 a | 0.472 |
| E1 | Phloem | 3.59 ± 0.86 a | 3.89 ± 0.90 a | 6.06 ± 1.28 a | 0.199 |
| E2 | Phloem | 353.54 ± 19.14 a | 341.52 ± 20.21 a | 334.82 ± 21.44 a | 0.817 |
| F | Epidermis and mesophyll | 20.46 ± 11.26 a | 14.06 ± 8.02 a | 2.45 ± 2.45 a | 0.336 |
| G | Xylem | 0.00 ± 0.00 a | 1.89 ± 1.60 a | 0.00 ± 0.00 a | 0.337 |
| *Other variables* | | | | | |
| Mean duration of pd (sec) | Epidermis and mesophyll | 4.10 ± 0.10 b | 4.46 ± 0.05 a | 4.15 ± 0.17 ab | **0.048** |

**Note.** The data were statistically analyzed by ANOVA followed by Tukey’s post hoc test. The *P* value is the global *P* value of ANOVA in this table. For the same row values by different lowercase letters indicate significant differences at the 0.05 level.

**B.** Means ± SEM of EPG variables for dsGFP, dsTPS and dsTRE treated red *A. pisum* feeding on *Vicia faba* after 48 h, during 8 h.

| Variables | Related to | dsGFP (n = 17) | dsTPS (n = 15) | dsTRE (n = 16) | *P* value |
| --- | --- | --- | --- | --- | --- |
| *Number of events of each waveform, per insect*.* | | | | | |
| np | All tissues | 10.41 ± 1.62 a | 9.40 ± 2.24 a | 6.63 ± 1.58 a | 0.313 |
| C | All tissues | 12.94 ± 1.66 a | 11.93 ± 2.22 a | 10.63 ± 1.63 a | 0.665 |
| E1 | Phloem | 3.06 ± 0.49 a | 3.53 ± 0.68 a | 4.63 ± 1.29 a | 0.437 |
| E2 | Phloem | 2.71 ± 0.43 a | 2.80 ± 0.38 a | 3.94 ± 0.84 a | 0.265 |
| F | Epidermis and mesophyll | 0.47 ± 0.15 a | 0.00 ± 0.00 b | 0.63 ± 0.18 a | **0.009** |
| G | Xylem | 0.41 ± 0.17 a | 0.13 ± 0.09 a | 0.31 ± 0.20 a | 0.486 |
| pd | Epidermis and mesophyll | 91.53 ± 15.25 a | 115.07 ± 15.19 a | 109.31 ± 19.33 a | 0.582 |
| probes | All tissues | 10.24 ± 1.59 a | 9.40 ± 2.24 a | 6.69 ± 1.61 a | 0.352 |
| *Total duration events of each waveform, per insects (min)**.* | | | | | |
| np | Epidermis and mesophyll | 19.86 ± 5.58 a | 20.52 ± 6.8 a | 16.05 ± 6.66 a | 0.866 |
| C | Epidermis and mesophyll | 143.63 ± 19.93 a | 122.86 ± 14.93 a | 122.05 ± 17.25 a | 0.615 |
| *E1* | *Phloem* | *3.55 ± 0.68 a* | *3.32 ± 0.89 a* | *3.49 ± 0.94 a* | *0.981* |
| E2 | Phloem | 239.74 ± 30.40 b | 327.48 ± 20.25 a | 241.15 ± 26.38 b | **0.039** |
| F | Epidermis and mesophyll | 44.44 ± 19.29 ab | 0.00 ± 0.00 b | 71.79 ± 23.00 a | **0.025** |
| G | Xylem | 28.78 ± 16.15 | 5.82 ± 4.19 | 25.48 ± 20.00 | 0.536 |
| *Other variables* | | | | | |
| Mean duration of pd (sec) | Epidermis and mesophyll | 4.10 ± 0.12 | 4.08 ± 0.09 | 4.34 ± 0.20 | 0.381 |

**Note.** The data were statistically analyzed by one-way ANOVA followed by Tukey’s post hoc test. The *P* value is the global *P* value of ANOVA in this table. For the same row values by different lowercase letters indicate significant differences at the 0.05 level.

**C.** Means ± SEM of EPG variables for dsGFP, dsTPS and dsTRE treated green *A. pisum* feeding on *Vicia faba* after 24 h, during 8 h.

| Variables | Related to | dsGFP (n = 16) | dsTPS (n = 16) | dsTRE (n = 16) | *P* value |
| --- | --- | --- | --- | --- | --- |
| *Number of events of each waveform, per insect*.* | | | | | |
| np | All tissues | 10.06 ± 2.28 a | 6.56 ± 1.41 a | 10.69 ± 1.37 a | 0.207 |
| C | All tissues | 13.69 ± 2.22 a | 10.19 ± 1.96 a | 16.50 ± 1.73 a | 0.089 |
| E1 | Phloem | 5.75 ± 0.91 a | 4.44 ± 0.98 a | 8.25 ± 1.43 a | 0.063 |
| E2 | Phloem | 4.38 ± 0.69 a | 3.88 ± 0.96 a | 5.63 ± 0.72 a | 0.290 |
| F | Epidermis and mesophyll | 0.19 ± 0.10 a | 0.19 ± 0.10 a | 0.13 ± 0.09 a | 0.868 |
| G | Xylem | 0.00 ± 0.00 a | 0.00 ± 0.00 a | 0.00 ± 0.00 a | 0.195 |
| pd | Epidermis and mesophyll | 152.94 ± 17.90 a | 132.63 ± 20.72 a | 156.94 ± 15.68 a | 0.603 |
| probes | All tissues | 10.06 ± 2.28 a | 6.56 ± 1.41 a | 10.63 ± 1.34 a | 0.210 |
| *Total duration events of each waveform, per insects (min)**.* | | | | | |
| np | Epidermis and mesophyll | 16.00 ± 5.51 a | 6.64 ± 1.79 a | 13.02 ± 3.25 a | 0.222 |
| C | Epidermis and mesophyll | 166.62 ± 15.69 ab | 131.24 ± 18.13 b | 205.14 ± 25.76 a | **0.046** |
| E1 | Phloem | 7.82 ± 1.96 a | 4.54 ± 1.20 a | 11.09 ± 2.76 a | 0.094 |
| E2 | Phloem | 279.94 ± 19.39 ab | 327.60 ± 18.32 a | 246.52 ± 21.78 b | **0.021** |
| F | Epidermis and mesophyll | 11.99 ± 6.61 a | 9.98 ± 5.67 a | 7.82 ± 5.46 a | 0.884 |
| G | Xylem | 0.00 ± 0.00 a | 0.00 ± 0.00 a | 0.00 ± 0.00 a | 0.337 |
| *Other variables* | | | | | |
| Mean duration of pd (sec) | Epidermis and mesophyll | 4.11 ± 0.08 a | 4.36 ± 0.10 a | 4.26 ± 0.08 a | 0.139 |

**Note.** The data were statistically analyzed by one-way ANOVA followed by Tukey’s post hoc test. The *P* value is the global *P* value of ANOVA in this table. For the same row values by different lowercase letters indicate significant differences at the 0.05 level.

**D.** Means ± SEM of EPG variables for dsGFP, dsTPS and dsTRE treated green *A. pisum* feeding on *Vicia faba* after 48 h, during 8 h.

| Variables | Related to | dsGFP (n = 17) | dsTPS (n = 16) | dsTRE (n = 15) | *P* value |
| --- | --- | --- | --- | --- | --- |
| *Number of events of each waveform, per insect*.* | | | | | |
| np | All tissues | 11.24 ± 2.56 a | 8.81 ± 1.33 a | 8.47 ± 1.78 a | 0.561 |
| C | All tissues | 14.71 ± 2.71 a | 11.69 ± 1.68 a | 12.53 ± 1.85 a | 0.591 |
| E1 | Phloem | 3.53 ± 0.61 a | 4.63 ± 0.76 a | 4.20 ± 0.87 a | 0.570 |
| E2 | Phloem | 3.35 ± 0.56 a | 3.88 ± 0.64 a | 3.87 ± 0.82 a | 0.815 |
| F | Epidermis and mesophyll | 0.82 ± 0.15 a | 0.31 ± 0.12 b | 0.73 ± 0.18 ab | **0.049** |
| G | Xylem | 0.24 ± 0.14 a | 0.13 ± 0.09 a | 0.13 ± 0.09 a | 0.722 |
| pd | Epidermis and mesophyll | 92.06 ± 13.98 a | 84.50 ± 9.60 a | 101.93 ± 16.77 a | 0.676 |
| probes | All tissues | 11.18 ± 2.53 a | 8.75 ± 1.33 a | 8.40 ± 1.77 a | 0.555 |
| *Total duration events of each waveform, per insects (min)**.* | | | | | |
| np | Epidermis and mesophyll | 29.86 ± 10.83 a | 18.35 ± 4.54 a | 12.28 ± 4.51 a | 0.255 |
| C | Epidermis and mesophyll | 126.43 ± 15.23 a | 115.53 ± 8.81 a | 123.27 ± 16.38 a | 0.847 |
| E1 | Phloem | 2.83 ± 0.45 b | 5.64 ± 1.11 a | 3.17 ± 0.64 b | **0.027** |
| E2 | Phloem | 223.94 ± 26.54 b | 310.89 ± 16.09 a | 269.29 ± 27.00 b | **0.040** |
| F | Epidermis and mesophyll | 90.75 ± 22.95 a | 23.91 ± 10.40 b | 65.81 ± 15.57 ab | **0.029** |
| G | Xylem | 7.94 ± 5.03 a | 4.03 ± 4.02 a | 6.19 ± 4.78 a | 0.834 |
| *Other variables* | | | | | |
| Mean duration of pd (sec) | Epidermis and mesophyll | 4.77 ± 0.20 a | 4.25 ± 0.08 b | 4.57 ± 0.05 ab | **0.028** |

**Note.** The data were statistically analyzed by one-way ANOVA followed by Tukey’s post hoc test. The *P* value is the global *P* value of ANOVA in this table. For the same row values by different lowercase letters indicate significant differences at the 0.05 level.

**E.** Means ± SEM of EPG variables for normal diet (CK), high trehalose diet (T100) and high glucose diet (G100) treated red *A. pisum* feeding on *Vicia faba* after 24 h, during 8 h.

| Variables | Related to | CK (n = 17) | T100 (n = 16) | G100 (n = 14) | *P* value |
| --- | --- | --- | --- | --- | --- |
| *Number of events of each waveform, per insect*.* | | | | | |
| np | All tissues | 7.24 ± 2.12 a | 6.13 ± 1.05 a | 7.86 ± 1.25 a | 0.750 |
| C | All tissues | 9.24 ± 2.59 b | 8.38 ± 0.99 b | 17.00 ± 2.31 a | **0.013** |
| E1 | Phloem | 4.76 ± 0.94 b | 2.25 ± 0.30 b | 10.71 ± 2.29 a | **0.000** |
| E2 | Phloem | 4.18 ± 0.81 a | 1.94 ± 0.25 b | 5.93 ± 0.70 a | **0.000** |
| F | Epidermis and mesophyll | 0.12 ± 0.08 a | 0.25± 0.11 a | 0.00 ± 0.00 a | 0.127 |
| G | Xylem | 0.53 ± 0.27 a | 0.63 ± 0.27 a | 0.86 ± 0.36 a | 0.739 |
| pd | Epidermis and mesophyll | 81.18 ± 11.65 b | 95.81 ± 17.65 b | 230.43 ± 28.90 a | **0.000** |
| probes | All tissues | 7.12 ± 2.12 a | 6.25 ± 1.03 a | 7.93 ± 1.25 a | 0.769 |
| *Total duration events of each waveform, per insects (min)**.* | | | | | |
| np | Epidermis and mesophyll | 8.69 ± 5.15 a | 18.37 ± 5.22 a | 10.57 ± 1.93 a | 0.284 |
| C | Epidermis and mesophyll | 98.57 ± 16.08 b | 121.06 ± 17.31 ab | 168.35 ± 22.11 a | **0.034** |
| E1 | Phloem | 6.73 ± 2.82 ab | 2.22 ± 0.46 b | 13.04 ± 4.63 a | **0.052** |
| E2 | Phloem | 358.00 ± 19.27 a | 254.49 ± 29.35 b | 205.80 ± 29.74 b | **0.001** |
| F | Epidermis and mesophyll | 0.93 ± 0.69 a | 27.47 ± 15.25 a | 0.00 ± 0.00 a | 0.059 |
| G | Xylem | 9.38 ± 7.84 a | 56.38 ± 27.01 a | 77.99 ± 31.98 a | 0.116 |
| *Other variables* | | | | | |
| Mean duration of pd (sec) | Epidermis and mesophyll | 3.90 ± 0.07 b | 4.30 ± 0.19 a | 3.24 ± 0.07 c | **0.000** |

**Note.** The data were statistically analyzed by one-way ANOVA followed by Tukey’s post hoc test. The *P* value is the global *P* value of ANOVA in this table. For the same row values by different lowercase letters indicate significant differences at the 0.05 level.

**F.** Means ± SEM of EPG variables for normal diet (CK), high trehalose diet (T100) and high glucose diet (G100) treated red *A. pisum* feeding on *Vicia faba* after 48 h, during 8 h.

| Variables | Related to | CK (n = 15) | T100 (n = 15) | G100 (n = 18) | *P* value |
| --- | --- | --- | --- | --- | --- |
| *Number of events of each waveform, per insect*.* | | | | | |
| np | All tissues | 11.47 ± 3.51 a | 7.07 ± 1.58 a | 9.44 ± 1.68 a | 0.445 |
| C | All tissues | 16.13 ± 3.52 a | 10.73 ± 2.19 a | 14.06 ± 1.93 a | 0.356 |
| E1 | Phloem | 4.80 ± 1.35 a | 3.87 ± 1.01 a | 5.33 ± 0.94 a | 0.632 |
| E2 | Phloem | 3.20 ± 0.76 a | 3.13 ± 0.73 a | 4.56 ± 0.75 a | 0.306 |
| F | Epidermis and mesophyll | 0.73 ± 0.27 a | 0.60 ± 0.19 a | 0.89 ±0.20 a | 0.639 |
| G | Xylem | 1.13 ± 0.36 a | 0.67 ± 0.23 ab | 0.17 ± 0.09 b | **0.022** |
| pd | Epidermis and mesophyll | 152.07 ± 23.36 a | 105.47 ± 22.03 a | 119.89 ± 20.47 a | 0.335 |
| probes | All tissues | 11.13 ± 3.52 a | 7.00 ± 1.59 a | 9.44 ± 1.68 a | 0.488 |
| *Total duration events of each waveform, per insects (min)**.* | | | | | |
| np | Epidermis and mesophyll | 22.70 ± 6.98 a | 10.94 ± 2.53 a | 14.45 ± 3.06 a | 0.191 |
| C | Epidermis and mesophyll | 177.54 ± 25.33 a | 124.15 ± 20.47 a | 148.63 ± 26.86 a | 0.346 |
| E1 | Phloem | 5.68 ± 2.21 a | 3.00 ± 0.72 a | 3.65 ± 0.65 a | 0.358 |
| E2 | Phloem | 166.76 ± 30.24 a | 233.37 ± 36.99 a | 244.15 ± 20.74 a | 0.143 |
| F | Epidermis and mesophyll | 55.23 ± 20.09 a | 54.90 ± 22.80 a | 57.32 ± 22.12 a | 0.628 |
| G | Xylem | 53.51 ± 17.49 a | 53.65 ± 26.67 a | 12.12 ± 8.65 a | 0.171 |
| *Other variables* | | | | | |
| Mean duration of pd (sec) | Epidermis and mesophyll | 3.76 ± 0.08 b | 4.29 ± 0.18 a | 3.78 ± 0.08 b | **0.005** |

**Note.** The data were statistically analyzed by one-way ANOVA followed by Tukey’s post hoc test. The *P* value is the global *P* value of ANOVA in this table. For the same row values by different lowercase letters indicate significant differences at the 0.05 level.

**G.** Means ± SEM of EPG variables for normal diet (CK), high trehalose diet (T100) and high glucose diet (G100) treated green *A. pisum* feeding on *Vicia faba* after 24 h, during 8 h.

| Variables | Related to | CK (n = 18) | T100 (n = 16) | G100 (n = 18) | *P* value |
| --- | --- | --- | --- | --- | --- |
| *Number of events of each waveform, per insect*.* | | | | | |
| np | All tissues | 9.39 ± 0.98 a | 8.81 ± 1.62 a | 6.67 ± 1.70 a | 0.377 |
| C | All tissues | 14.33 ± 1.38 a | 12.94 ± 1.92 a | 10.28 ± 1.94 a | 0.250 |
| E1 | Phloem | 7.17 ± 1.51 a | 5.31 ± 0.93 a | 3.67 ± 0.75 a | 0.090 |
| E2 | Phloem | 5.44 ± 1.03 a | 4.25 ± 0.70 a | 2.89 ± 0.59 a | 0.083 |
| F | Epidermis and mesophyll | 0.06 ± 0.06 a | 0.25 ± 0.14 a | 0.39 ± 0.14 a | 0.143 |
| G | Xylem | 0.17 ± 0.09 a | 0.13 ± 0.09 b a | 0.28 ± 0.11 a | 0.515 |
| pd | Epidermis and mesophyll | 156.72 ± 22.97 a | 147.88 ± 20.31 a | 127.61 ± 17.68 a | 0.581 |
| probes | All tissues | 9.44 ± 0.95 a | 8.94 ± 1.64 a | 7.06 ± 1.75 a | 0.479 |
| *Total duration events of each waveform, per insects (min)**.* | | | | | |
| np | Epidermis and mesophyll | 16.36 ± 3.77 a | 13.28 ± 1.97 a | 9.19 ± 3.07 a | 0.258 |
| C | Epidermis and mesophyll | 170.06 ± 16.73 a | 175.19 ± 18.15 a | 127.03 ± 16.37 a | 0.098 |
| E1 | Phloem | 10.32 ± 3.09 a | 6.19 ± 1.20 a | 5.51 ± 1.37 a | 0.224 |
| E2 | Phloem | 283.49 ± 18.08 a | 263.23 ± 19.59 a | 290.53 ± 19.41 a | 0.589 |
| F | Epidermis and mesophyll | 1.02 ± 1.02 a | 19.73 ± 10.64 a | 37.58 ± 16.64 a | 0.083 |
| G | Xylem | 3.04 ± 1.69 a | 3.17 ± 2.19 a | 10.14 ± 7.24 a | 0.459 |
| *Other variables* | | | | | |
| Mean duration of pd (sec) | Epidermis and mesophyll | 3.80 ± 0.06 b | 4.44 ± 0.18 a | 3.70 ± 0.08 b | **0.000** |

**Note.** The data were statistically analyzed by one-way ANOVA followed by Tukey’s post hoc test. The *P* value is the global *P* value of ANOVA in this table. For the same row values by different lowercase letters indicate significant differences at the 0.05 level.

**H.** Means ± SEM of EPG variables for normal diet (CK), high trehalose diet (T100) and high glucose diet (G100) treated green *A. pisum* feeding on *Vicia faba* after 48 h, during 8 h.

| Variables | Related to | CK (n = 15) | T100 (n = 18) | G100 (n = 18) | *P* value |
| --- | --- | --- | --- | --- | --- |
| *Number of events of each waveform, per insect*.* | | | | | |
| np | All tissues | 7.93 ± 1.60 a | 12.83 ± 2.88 a | 7.06 ± 1.07 a | 0.102 |
| C | All tissues | 11.87 ± 1.91 a | 16.56 ± 2.94 a | 10.65 ± 1.19 a | 0.114 |
| E1 | Phloem | 4.67 ± 1.00 a | 4.33 ± 1.03 a | 3.71 ± 0.85 a | 0.703 |
| E2 | Phloem | 3.93 ± 0.81 a | 3.22 ± 0.75 a | 3.24 ± 0.62 a | 0.697 |
| F | Epidermis and mesophyll | 0.60 ± 0.16 a | 1.00 ± 0.23 a | 0.59 ± 0.19 a | 0.216 |
| G | Xylem | 0.53 ± 0.24 a | 0.28 ± 0.14 a | 0.65 ± 0.24 a | 0.467 |
| pd | Epidermis and mesophyll | 120.00 ± 15.77 a | 119.67 ± 18.42 a | 135.00 ± 19.69 a | 0.910 |
| probes | All tissues | 7.87 ± 1.60 a | 12.83 ± 2.88 a | 7.06 ± 1.07 a | 0.100 |
| *Total duration events of each waveform, per insects (min)**.* | | | | | |
| np | Epidermis and mesophyll | 11.46 ± 2.84 a | 18.61 ± 7.77 a | 11.05 ± 2.56 a | 0.191 |
| C | Epidermis and mesophyll | 151.50 ± 15.71 a | 155.11 ± 32.67 a | 145.23 ± 17.96 a | 0.561 |
| E1 | Phloem | 5.11 ± 1.06 a | 4.82 ± 1.45 a | 3.95 ± 1.13 a | 0.721 |
| E2 | Phloem | 186.20 ± 31.04 a | 169.82 ± 24.00 a | 193.72 ± 31.44 a | 0.658 |
| F | Epidermis and mesophyll | 87.36 ± 25.61 a | 108.38 ± 28.68 a | 80.10 ± 27.84 a | 0.679 |
| G | Xylem | 39.67 ± 26.11 a | 23.61 ± 11.97 a | 45.95 ± 20.94 a | 0.738 |
| *Other variables* | | | | | |
| Mean duration of pd (sec) | Epidermis and mesophyll | 3.99 ± 0.10 b | 3.78 ± 0.05 b | 4.43 ± 0.18 a | **0.003** |

**Note.** The data were statistically analyzed by one-way ANOVA followed by Tukey’s post hoc test. The *P* value is the global *P* value of ANOVA in this table. For the same row values by different lowercase letters indicate significant differences at the 0.05 level.

**2. Figures**


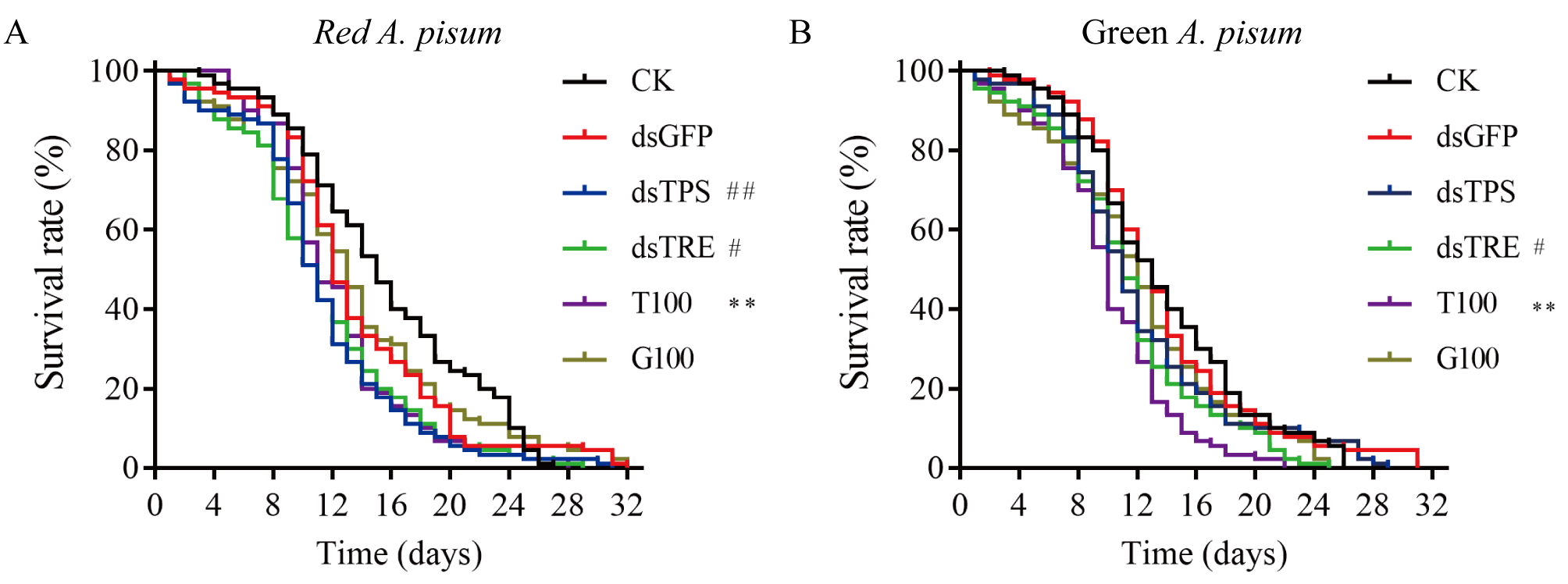


**Figure S1. The survival curves for *A. pisum*.** The survival curves for red (**A**) and green (**B**) *A. pisum*; RNAi of *GFP* (**dsGFP**), RNAi of *TPS* (**dsTPS**), RNAi of *TRE* (**dsTRE**), high trehalose diet (**T100**), high glucose diet (**G100**) and normal diet (**CK**). The Kaplan-Meier survival log-rank analysis was used to compare the overall survivals of the untreated aphids (CK) with the treated aphids (T100 and G100) and dsGFP-treated aphids. ^**^*P*<0.01 vs. CK; ^#^*P*<0.05 and ^##^*P*<0.01 vs. dsGFP. Edited in GraphPad Prism version 7.00 (https://www.graphpad.com/scientific-software/prism/).


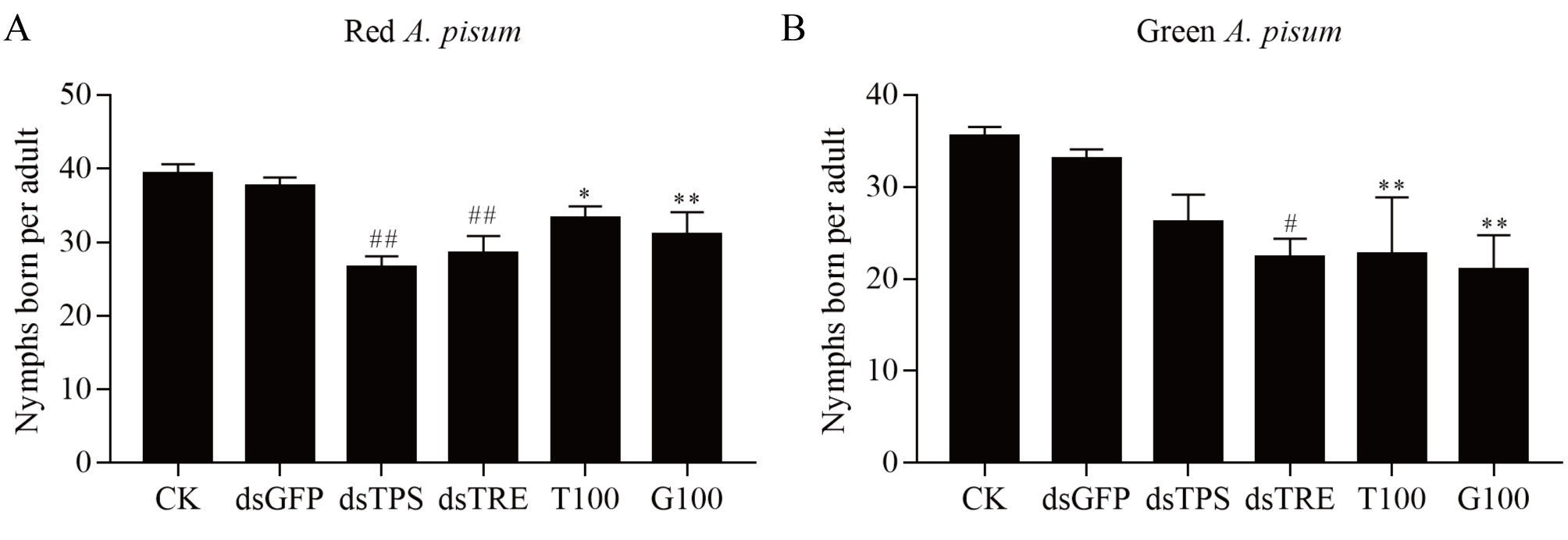


**Figure S2. The reproduction for *A. pisum*.** Nymphs born per red (**A**) and green (**B**) *A. pisum* adult. RNAi of *GFP* (**dsGFP**), RNAi of *TPS* (**dsTPS**), RNAi of *TRE* (**dsTRE**), high trehalose diet (**T100**), high glucose diet (**G100**) and normal diet (**CK**). All data were represented as means ± SME of three replicates and were analyzed using ANOVA followed by the Tukey’s post hoc test. ^*^*P*<0.01 and ^**^*P*<0.01 vs. CK; ^#^*P*<0.05 and ^##^*P*<0.01 vs. dsGFP. Edited in GraphPad Prism version 7.00 (https://www.graphpad.com/scientific-software/prism/).


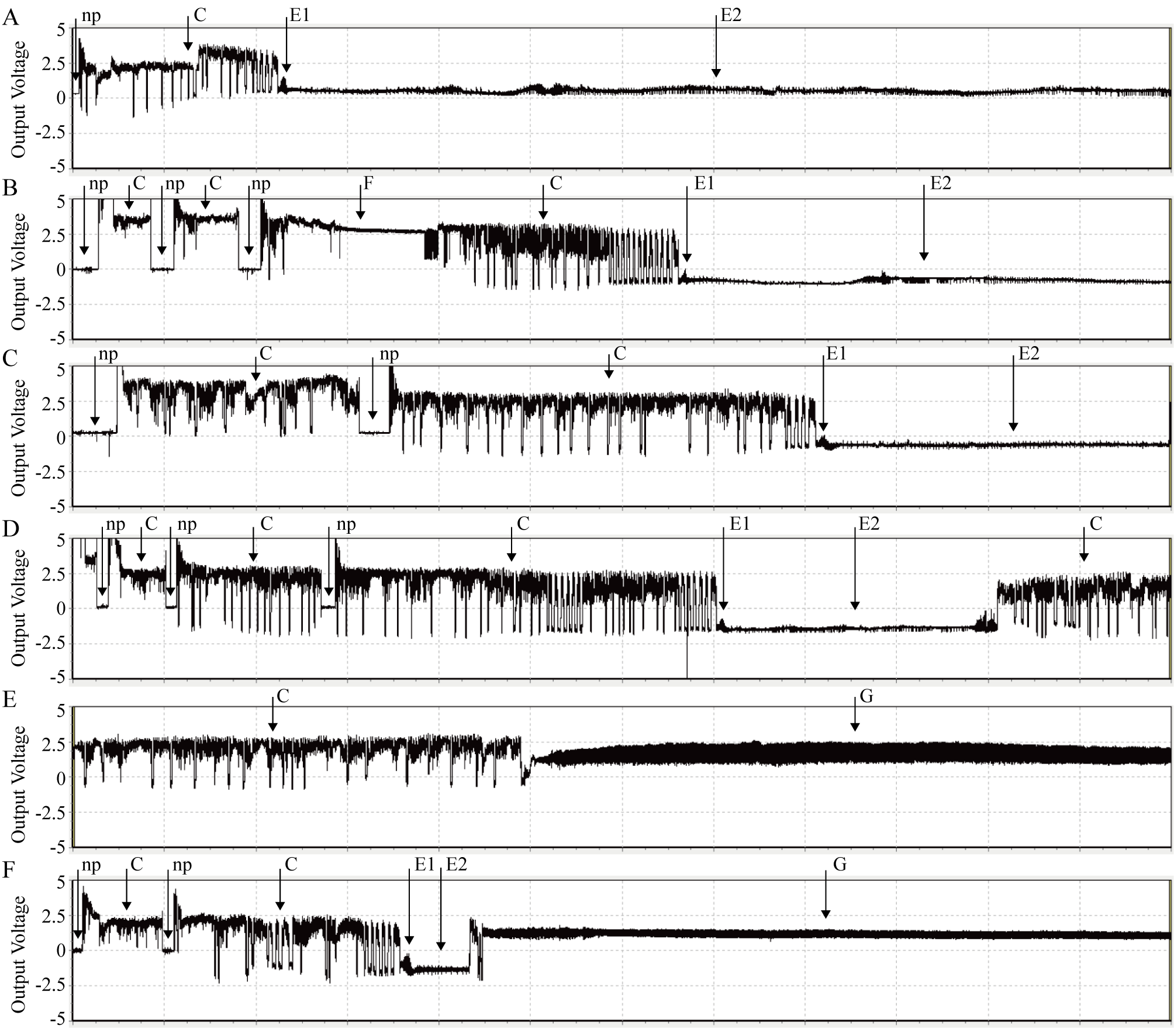


**Figure S3. Representative EPG recordings of each treatment red *A. pisum* and control *A. pisum* on *Vicia faba* after 24 h, during 1 h.** A. normal diet (**CK**), B. RNAi of *GFP* (**dsGFP**), C. RNAi of *TPS* (**dsTPS**), D. RNAi of *TRE* (**dsTRE**), E. high trehalose diet (**T100**), F. high glucose diet (**G100**), np. non-probing, C. waveform for intercellular apoplastic stylet pathway, E1. waveform for phloem salivation, E2. waveform for phloem ingestion, F. waveform for derailed stylet mechanics, G. waveform for xylem ingestion. Edited in Stylet+ version av01.30 (https://www.epgsystems.eu/).


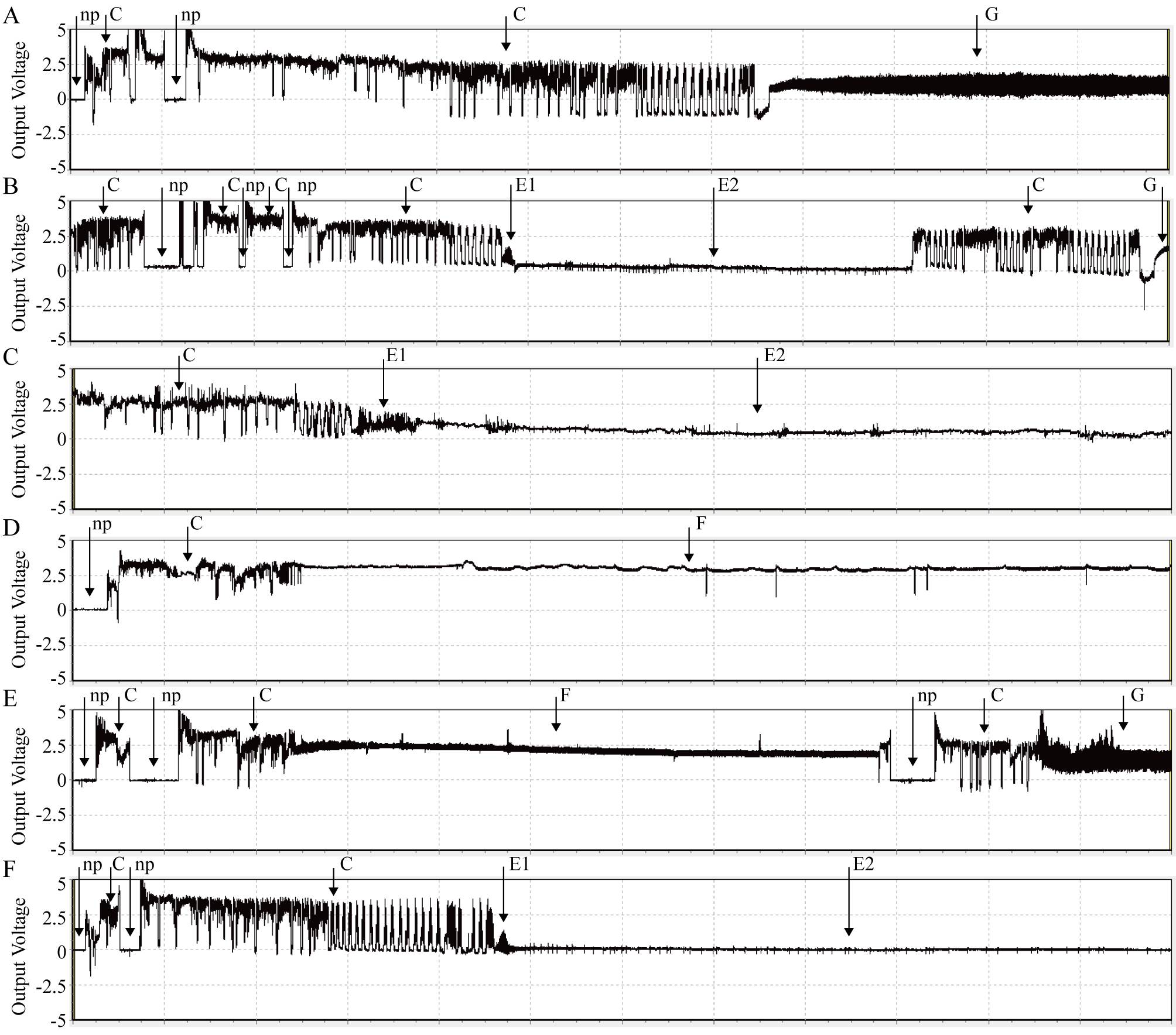


**Figure S4. Representative EPG recordings of each treatment red *A. pisum* and control *A. pisum* on *Vicia faba* after 48 h, during 1 h.** A. normal diet (**CK**), B. RNAi of *GFP* (**dsGFP**), C. RNAi of *TPS* (**dsTPS**), D. RNAi of *TRE* (**dsTRE**), E. high trehalose diet (**T100**), F. high glucose diet (**G100**), np. non-probing, C. waveform for intercellular apoplastic stylet pathway, E1. waveform for phloem salivation, E2. waveform for phloem ingestion, F. waveform for derailed stylet mechanics, G. waveform for xylem ingestion. Edited in Stylet+ version av01.30 (https://www.epgsystems.eu/).


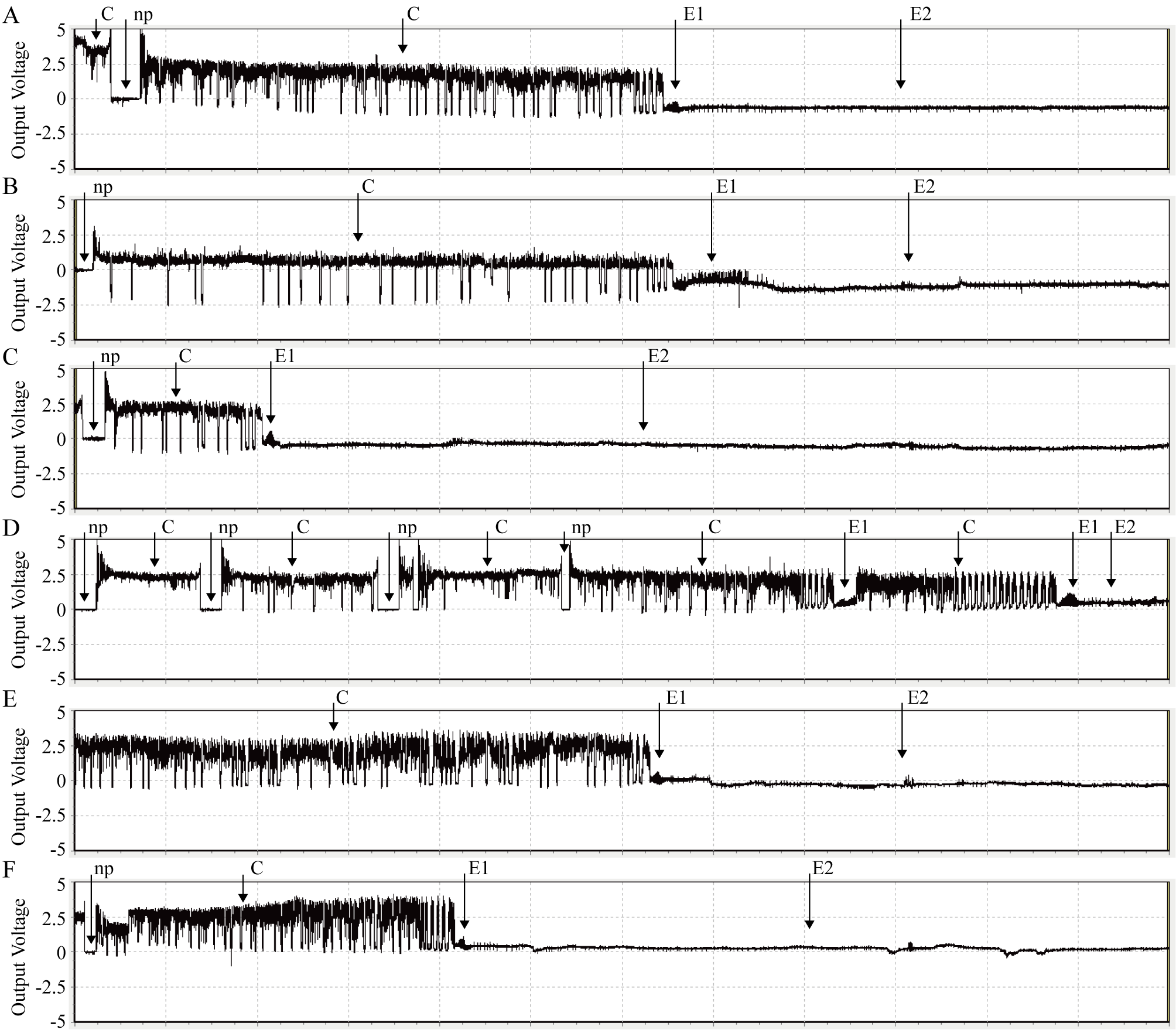


**Figure S5. Representative EPG recordings of each treatment green *A. pisum* and control *A. pisum* on *Vicia faba* after 24 h, during 1 h.** A. normal diet (**CK**), B. RNAi of *GFP* (**dsGFP**), C. RNAi of *TPS* (**dsTPS**), D. RNAi of *TRE* (**dsTRE**), E. high trehalose diet (**T100**), F. high glucose diet (**G100**), np. non-probing, C. waveform for intercellular apoplastic stylet pathway, E1. waveform for phloem salivation, E2. waveform for phloem ingestion, F. waveform for derailed stylet mechanics, G. waveform for xylem ingestion. Edited in Stylet+ version av01.30 (https://www.epgsystems.eu/).


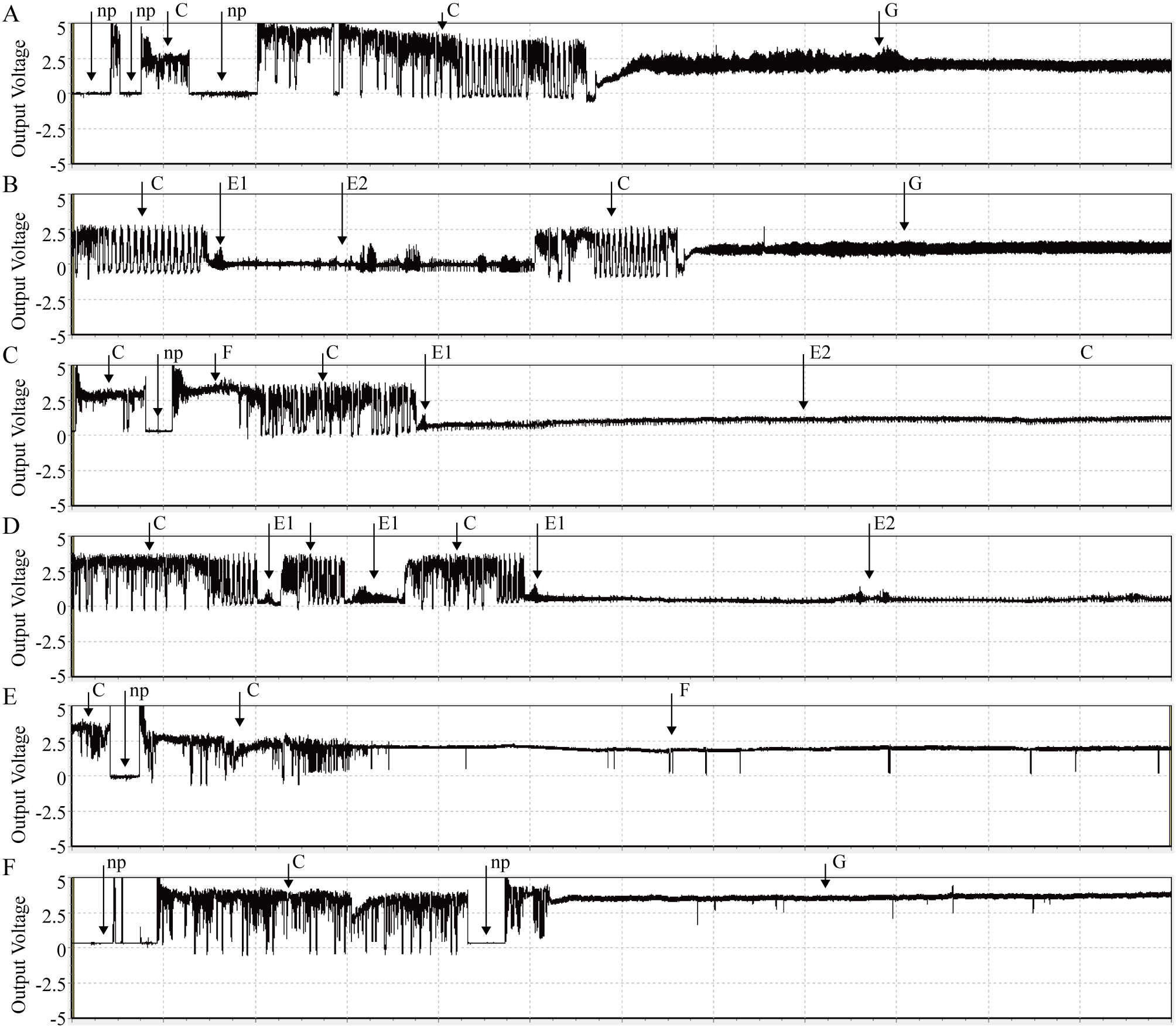


**Figure S6. Representative EPG recordings of each treatment green *A. pisum* and control *A. pisum* on *Vicia faba* after 48 h, during 1 h.** A. normal diet (**CK**), B. RNAi of *GFP* (**dsGFP**), C. RNAi of *TPS* (**dsTPS**), D. RNAi of *TRE* (**dsTRE**), E. high trehalose diet (**T100**), F. high glucose diet (**G100**), np. non-probing, C. waveform for intercellular apoplastic stylet pathway, E1. waveform for phloem salivation, E2. waveform for phloem ingestion, F. waveform for derailed stylet mechanics, G. waveform for xylem ingestion. Edited in Stylet+ version av01.30 (https://www.epgsystems.eu/).
